# Cell Labeling with 15-YNE Is Useful for Tracking Protein Palmitoylation and Metabolic Lipid Flux in the Same Sample

**DOI:** 10.3390/molecules30020377

**Published:** 2025-01-17

**Authors:** Nadine Merz, Karin Schilling, Dominique Thomas, Lisa Hahnefeld, Sabine Grösch

**Affiliations:** 1 Goethe University Frankfurt, Institute of Clinical Pharmacology, Faculty of Medicine, Theodor Stern Kai 7, 60590 Frankfurt am Main, Germany; n.merz@med.uni-frankfurt.de (N.M.); schilling@med.uni-frankfurt.de (K.S.); thomas@med.uni-frankfurt.de (D.T.); hahnefeld@med.uni-frankfurt.de (L.H.); 2 Fraunhofer Institute for Translational Medicine and Pharmacology ITMP, Theodor-Stern-Kai 7, 60596 Frankfurt am Main, Germany; 3 Fraunhofer Cluster of Excellence for Immune-Mediated Diseases CIMD, Theodor-Stern-Kai 7, 60596 Frankfurt am Main, Germany

**Keywords:** Cy5.5-azide, biotin, protein palmitoylation, alkyne, click reaction, Linex, LC-HRMS

## Abstract

Protein S-palmitoylation is the process by which a palmitoyl fatty acid is attached to a cysteine residue of a protein via a thioester bond. A range of methodologies are available for the detection of protein S-palmitoylation. In this study, two methods for the S-palmitoylation of different proteins were compared after metabolic labeling of cells with 15-hexadecynoic acid (15-YNE) to ascertain their relative usefulness. It was hypothesized that labeling cells with a traceable lipid would affect lipid metabolism and the cellular lipidome. In this study, we developed a method to track 15-YNE incorporation into lipids using liquid chromatography high-resolution mass spectrometry (LC-HRMS) as well as protein palmitoylation in the same sample. We observed a time- and concentration-dependent S-palmitoylation of calnexin and succinate dehydrogenase complex flavoprotein subunit A (SDHA) depending on the cell type. The detection of S-palmitoylation with a clickable fluorophore or biotin azide followed by immunoprecipitation is shown to be equally useful. 15-YNE was observed to be incorporated into a wide array of lipid classes during the process, yet it did not appear to modify the overall lipid composition of the cells. In conclusion, we show that 15-YNE is a useful tracer to detect both protein S-palmitoylation and lipid metabolism in the same sample.

## 1. Introduction

**Figure 1:**
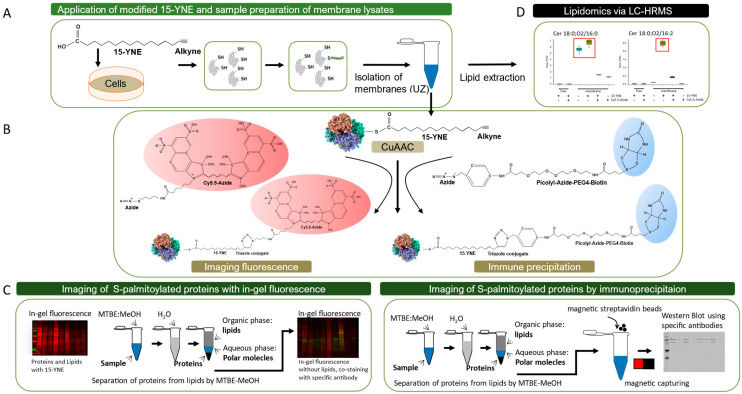
Schematic diagram of the workflow and analysis methods. After treatment of cells with 15-YNE, cells were harvested and membranes were isolated by centrifugation (**A**). Membrane fractions were either clicked to Cy5.5-azide or Picolyl-azide-PEG4-biotin (biotin-azide) (**B**). Palmitoylated proteins were detected either by fluorescence imaging or via immunoprecipitation and Western blot analysis (**C**). Lipids were extracted from membrane fractions and analyzed via LC-HRMS (**D**). The library match of fatty acid 16:2 (FA16:2) is an isomer to 15-YNE and considered to be 15-YNE throughout the manuscript.

Post-translational modifications of proteins are manifold and include phosphorylation, ubiquitination, glycosylation, SUMOylation, and lipidation. They can influence protein stability and activity and the subcellular transport or location of proteins. Lipidated proteins have a higher binding affinity to cellular membranes and show enhanced protein–protein interactions [1]. Protein lipidation includes the attachment of fatty acids (myristate, palmitate, and palmitoleate are the most common), phospholipids, cholesterol, farnesyl, or geranylgeranyl to proteins [2]. The attachment of fatty acids to proteins occurs at cysteine, serine, or lysine residues. The most prevalent type of protein lipidation is S-palmitoylation, which involves the covalent attachment of palmitate to a cysteine residue. S-palmitoylation is catalyzed by the Asp–His–His–Cys-rich domain (DHHC) palmitoyl acyl transferase (PAT) family, of which 23 isoforms are known in mammals [3]. Protein palmitoylation is a reversible modification and can be reversed by various depalmitoylating enzymes, including palmitoyl esterase, acyl protein thioesterase (APT1/APT2), and protein palmitoyl thioesterase (PPT1/PPT2) [4]. Several methods have been developed to detect protein S-palmitoylation, including the acyl–biotin exchange (ABE), the acyl-resin-assisted capture (acyl-RAC), and the metabolic labeling method. The ABE-based method detects palmitoylated proteins by replacing the thioester-linked palmitoyl modifications with a stable biotin tag. The limitations of this method are based on the fact that only cysteine palmitoylation (S-palmitoylation) can be detected and not the palmitoylation at serine, threonine, or lysine residues. Furthermore, as the assay involves multiple steps, proteins may be degraded or lost during the protocol, giving misleading results [5]. The acyl-resin-assisted capture (acyl-RAC) method replaces the thioester-linked palmitoyl modifications with a thiol-reactive resin that can be used for the direct pull-down of labeled proteins. The limitations of this method include an incomplete blockade of cysteine residues prior to hydroxylamine cleavage that results in binding of thiol-reactive resin to non-acylated proteins, leading to false-positive results. Additionally, this technique is unable to distinguish between different fatty acids that can be covalently bound to the cysteine residues [6]. Detection of S-palmitoylation of proteins by the metabolic labeling method has the advantage that it can be used for the detection of dynamic changes and turnover rates [7]. For metabolic labeling, either radiolabeled fatty acids or clickable fatty acids can be used. The click-chemistry-based method comprises the biorthogonal Cu(I)-catalyzed cycloaddition of an azide-containing fluorophore or biotin to 15-hexadecynoic acid (15-YNE, a palmitic acid that contains an alkYNE group at the ω position) [7,8]. Treatment with a clickable fatty acid, for example with palmitic acid, takes place in living cells whereas the click reaction is performed in vitro due to the cytotoxic activity of Cu-SO_4_ (Figure 1). Palmitic acid is supplied in cell culture media and taken up either by diffusion or by membrane-located transport proteins (solute carrier family 27 (SLC27) or fatty acid transport proteins 1–6 (FATPs 1–6) and fatty acid translocase (FAT)/CD36) [9]. Uptake by FATPs is coupled to esterification of fatty acids with coenzyme A mediated by long-chain acyl CoA synthetase (Acsl) [10]. After intracellular uptake, 15-YNE is either used for protein palmitoylation or incorporated into lipids. The palmitoylation of proteins with 15-YNE can be detected by either a clickable fluorophore such as Cyanine 5.5-azide (Cy5.5- azide) or biotin-azide.

Fatty acids with an azide or alkyne group at the ω position have been shown to elongate and desaturate when incorporated into various lipids [11,12]. Thus, 15-YNE is also useful for detecting lipidomic changes and metabolism in cells. However, it must be excluded that the labeling of cells with 15-YNE affects the lipidome itself. Therefore, we analyzed the lipidome of cells after 15-YNE treatment by liquid chromatography high-resolution mass spectrometry (LC-HRMS). The lipidomic data were scrutinized by the interactive bioinformatics web application LINEX2 (Lipid Network Explorer), which facilitates the analysis of lipidomic data by the introduction of graph-theoretical and network-topological visualizations [13]. LINEX2 combines enzyme-catalyzed lipid transformations with correlation algorithms and statistical properties, which are depicted in an interactive network. It visualizes biochemical connections between lipid classes, taking account of the multi-specificity of metabolic reactions by lipid enzymes via an optimized enrichment graph algorithm (a network extension to lipid species based on curated lipid class reactions from the Rhea [14] and Reactome [15] databases) for the interpretation of lipidomic data. LINEX2 was used to compare 15-YNE-treated versus non-treated cells to uncover potential dysregulations of the lipid metabolism by deciphering lipid metabolism and regulatory mechanisms between lipid species [16,17].

In this study, we demonstrated the efficacy of labeling cells with 15-YNE for the detection of protein palmitoylation and the tracking of its incorporation into lipids within a single sample. Utilizing Cyanine 5.5-azide (Cy5.5-azide) or biotin-azide for the click reaction, we elucidated the parallels and discrepancies between the two labeling methodologies along with their respective advantages and disadvantages in terms of detection.

## 2. Results

We investigated the palmitoylation of proteins by metabolic labeling and click chemistry. In this study, we aimed to provide a comprehensive evaluation of the merits and drawbacks of the various azide chemicals that can be employed for the visualization of protein palmitoylation. In addition, we tracked the distribution of 15-YNE, the metabolic labeling agent utilized in this study, within cellular fractions and its incorporation into lipids using LC-HRMS. This approach was employed to ascertain whether treatment of cells with 15-YNE exerts an influence on the lipid rheostat. This is of particular relevance, as alterations in the lipid composition of membranes may also impact the membrane localization of lipidated proteins. The workflow of our analysis is depicted in Figure 1. The detailed method is outlined in the section titled “Materials and Methods”.

### 2.1. Comparison of Fluorescence and Immunoprecipitation Detection of Protein Palmitoylation

**Figure 2:**
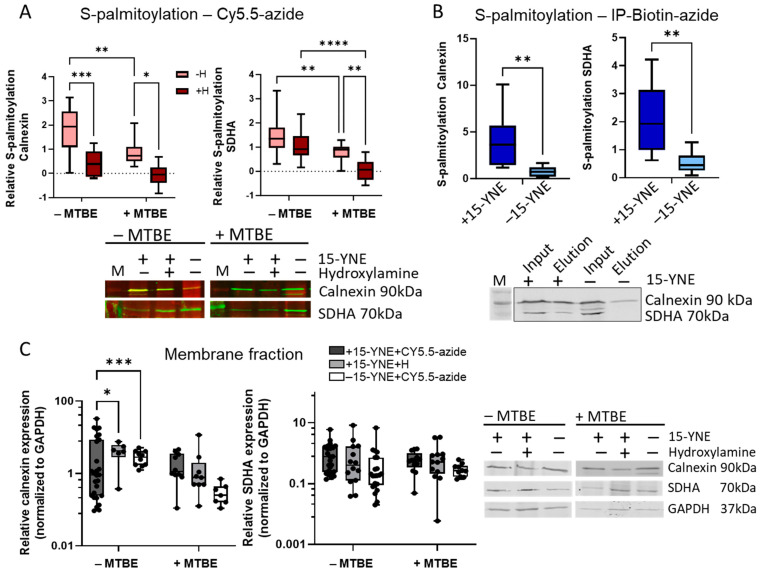
Detection of S-palmitoylated proteins. S-Palmitoylation of calnexin and SDHA in +15-YNE-treated cells. (**A**) 15-YNE was coupled to Cy5.5-azide by click reaction. Thereafter, the samples underwent treatment with or without hydroxylamine (+H/–H). Subsequent to this, the samples were subjected to either MTBE/methanol treatment or no treatment. Calnexin and SDHA were detected by Western blot and protein palmitoylation was calculated by the following formula: S-palmitoylation $=\frac{\left(\frac{700}{800}\;nm\right)+15-YNE+Cy5.5-azide\;target\;protein}{\left(\frac{700}{800\;}nm\right)-15-YNE+H\;target\;protein\;background}$. (**B**) Protein S-palmitoylation of biotin/streptavidin immunoprecipitated samples was calculated by the ratio of the target antibody signal (at 700 nm or 800 nm, normalized to the full protein signal of the lane) vs. the respective, normalized target protein input amount (400 µg) from the same Western blot. (**C**) Analysis of relative protein expression of calnexin (left) and SDHA (right) from the same Western blots against the signal of GAPDH to provide assurance of equal loading of Western blots. Statistical significance was calculated with two-way ANOVA with a mixed-effects analysis and a post-hoc Tukey’s multiple comparison test, α = 0.05; * *p* < 0.05; ** *p* < 0.01; *** *p* < 0.001; **** *p* < 0.0001. The data are presented as interleaved box and whiskers, Min. to Max., showing all points.

We investigated the protein palmitoylation of two well-known S-palmitoylated proteins, calnexin and succinate dehydrogenase complex flavoprotein subunit A (SDHA), in human colon cancer cells (HCT-116) using two different detection methods [18,19]. On the one hand, we examined protein palmitoylation subsequent to metabolic labeling of cells with 15-YNE by appending a Cy5.5-azide to the alkine group of 15-YNE. On the other hand, we used biotin-azide and subsequent immunoprecipitation. The proteins were separated by Western blot. The 15-YNE-labeled proteins that were coupled to Cy5.5-azide were detected at a wavelength of 710 nm using the Odyssey^®^ M Imaging System (LI-COR). The specific antibodies against calnexin and SDHA were detected with a secondary antibody that was conjugated to IRDye 800CW (Figure 2A). We detected a higher co-staining signal when we separated clicked samples directly with SDS-polyacrylamid gelelektrophoresis (SDS-PAGE) and subsequently transferred the proteins to a nitrocellulose membrane. Treatment of samples with hydroxylamine (+/−H) did not result in the complete elimination of the co-staining signal, suggesting that background signals, which are not S-palmitoylated proteins, were also detected. Hydroxylamine has been shown to cleave the thioester bond of S-acetylated proteins rather than the ester bond that may form between 15-YNE and other cell metabolites, such as lipids. However, hydroxylamine has been shown to cleave pH-dependent proteins at Asn-Gly (NG) sites [20]. Therefore, it is important to note that the concentration of hydroxylamine used here (0.6 M) should not be exceeded as this may result in protein degradation. To eliminate the background signal of Cy5.5 in the samples, the clicked samples were treated with methyl tert-butyl ether (MTBE) and methanol to separate lipids and other cell compounds from proteins [21]. The application of MTBE/methanol treatment resulted in the separation of the clicked samples into three distinct phases, as illustrated in Figure 1. The upper phase is the organic phase, which contains lipids, while the lower phase is the aqueous phase, comprising polar molecules. At the base of the tube, a dense protein pellet was obtained. The protein pellet was resuspended and separated by SDS-PAGE, and S-palmitoylation was calculated using the co-staining signal related to the signal of the corresponding protein without Cy5.5 staining. Using this method, we observed a significantly lower S-palmitoylation signal compared with non-MTBE/methanol-treated samples (−MTBE), which completely vanished upon treatment with hydroxylamine. Therefore, when using 15-YNE for metabolic labeling and click chemistry, it is imperative to separate lipids from proteins before detecting S-palmitoylated proteins.

As an alternative detection method for S-palmitoylation after metabolic labeling with 15-YNE, we used biotin-azide for the click reaction. Again, the lipids were removed by MTBE/methanol treatment and the proteins were immunoprecipitated from the protein pellet using streptavidin beads (Figure 1). We detected calnexin and SDHA in the eluate of the immunoprecipitants in comparison to the input in +15-YNE-treated (+15-YNE) samples and in samples without 15-YNE (−15-YNE). The S-palmitoylation signal for calnexin and SDHA was significantly higher in +15-YNE-treated samples compared with −15-YNE samples (Figure 2B). The presence of a very low signal for calnexin and SDHA in the −15-YNE-treated samples might be attributable to the washing procedure prior to elution.

To exclude the possibility that the variations in S-palmitoylation of our proteins were influenced by the total protein amount of calnexin or SDHA in the membrane fraction of 15-YNE+/− and hydroxylamine +/− -treated samples, we correlated the expression of calnexin and SDHA with the housekeeping gene GAPDH (Figure 2C). While there was a significant difference in the relative expression level of calnexin between the +15-YNE+Cy5.5-azide samples and the +15-YNE+ H or −15-YNE+Cy5.5-azide samples, the relative expression level of calnexin in many +15-YNE +Cy5.5-azide samples was lower compared with the hydroxylamine-treated samples or the −15-YNE samples. This indicates that the differences in the S-palmitoylation signal in the hydroxylamine-treated samples cannot be explained by changes in the total amount of proteins in the samples but are due to depalmitoylation of the respective proteins. No significant differences in the relative protein expression level of SDHA could be detected in membrane lysates.

In summary, we detected S-palmitoylation of calnexin and SDHA after metabolic labeling with 15-YNE and clicking to Cy5.5 or biotin and subsequent Western blot analysis. Because 15-YNE incorporates into lipids as well, the fluorescence signal from S-palmitoylated proteins labeled with Cy5.5 is superimposed by the lipids. This could be overcome by precipitating proteins and extracting lipids with MTBE/methanol treatment. The same phenomenon was observed when 15-YNE was coupled to biotin and proteins were isolated via immunoprecipitation. To prevent overloading of the streptavidin-binding capacity of the beads with lipids, it is essential to separate proteins from lipids with MTBE/methanol prior to immunoprecipitation. It is important to note that both methods are equally effective for detecting protein palmitoylation.

### 2.2. Time and Concentration Dependency of Palmitoylation

**Figure 3:**
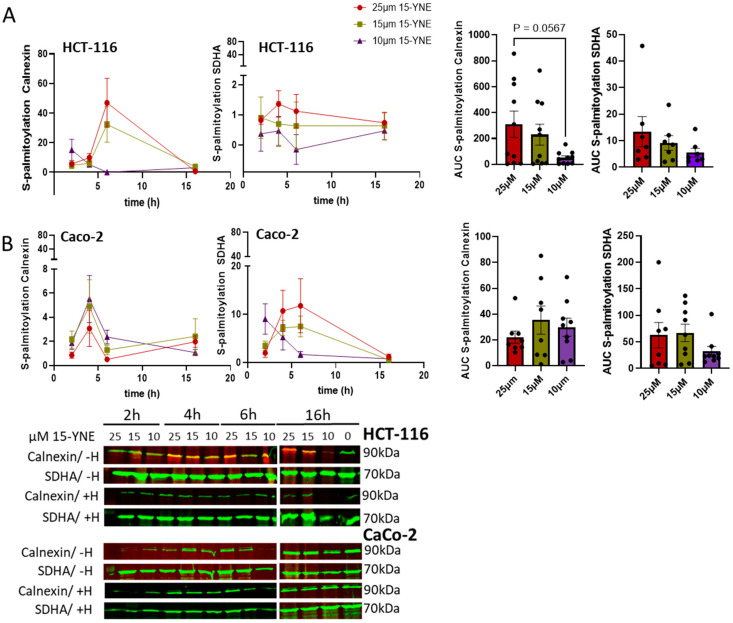
Time and concentration-dependent S-palmitoylation of calnexin and SDHA in HCT-116 and Caco-2 cells. HCT-116 cells (**A**) and Caco-2 cells (**B**) were treated with different concentrations of 15-YNE for 2, 4, 6, and 16 h. Thereafter, membrane extracts were isolated and clicked to Cy5.5-azide +/− hydroxylamine and proteins were subsequently separated on SDS-PAGE. S-palmitoylation of calnexin and SDHA was calculated by the following formula. S-palmitoylation $=\frac{\left(\frac{700}{800}\;nm\right)+15-YNE+Cy5.5-azide\;target\;protein}{\left(\frac{700}{800\;}nm\right)-15-YNE+H\;target\;protein\;background}$.

S-palmitoylation is a reversible process that can be reverted by palmitoyl esterase, acyl protein thioesterase (APT1/APT2), or protein palmitoyl thioesterase (PPT1/PPT2) [4]. The duration of S-palmitoylation varies depending on the protein and can last for only a few minutes or several hours [22]. We investigated the time and concentration-dependent incorporation of 15-YNE in two colon cancer cell lines, HCT-116 and Caco-2, to check whether there are cell-type-specific differences in protein palmitoylation. We used different 15-YNE concentrations (10, 15, and 25 µM) that were added to cell culture media for 2, 4, 6, or 16 h. Click reaction with Cy5.5-azide was performed with protein membrane lysates that were subsequently separated on SDS-PAGE and blotted onto a nitrocellulose membrane. Proteins that were palmitoylated with 15-YNE and clicked to Cy5.5-azide were detected at 710 nm. Specific antibodies against calnexin and SDHA together with a secondary antibody that was conjugated to IRDye 800CW were used for co-staining (Figure 3A,B). Palmitoylation of proteins was calculated using the co-staining signal related to the signal of the respective protein without Cy5.5 staining (see also the description in the Materials and Methods section). In HCT-116, a time and concentration-dependent 15-YNE palmitoylation of calnexin and SDHA could be detected with a maximum between 4 and 6 h using 25 µM 15-YNE (see Figure 3A,B). In Caco-2 cells, we also detected a time-dependent S-palmitoylation with 15-YNE, but there was no clear concentration dependency of palmitoylation with 15-YNE. Treatment with hydroxylamine reversed S-palmitoylation of proteins, as demonstrated in Figure 3.

The area under the curve (AUC) was calculated by the graph PadPrism 10.4 software. Statistical significance was calculated with one-way ANOVA with a mixed-effects analysis and a post-hoc Tukey’s multiple comparison test, α = 0.05.

In conclusion, S-palmitoylation of proteins occurs in a time- and concentration-dependent manner. However, the concentration dependence was not observed in Caco-2 cells, indicating that S-palmitoylation of proteins differs in different cell types and probably depends on the expression of ZDHHC enzymes as well as various palmitoyl esterases.

### 2.3. Tracking of 15-YNE in Different Cell Fractions

To investigate the distribution of 15-YNE in different cell fractions, we determined free 15-YNE using liquid chromatography high-resolution mass spectrometry (LC-HRMS). It was identified as C16 H28O2, which has the same molecular formula as hecadecadienoic acid (FA16:2). 15-YNE was determined in cell culture media directly after the addition of 25 µM 15-YNE (0 h), as well as in cell culture media, in membrane lysates, and in cytosol after a 4 h incubation time. Lipids from all fractions were extracted by organic solvent.

**Figure 4:**
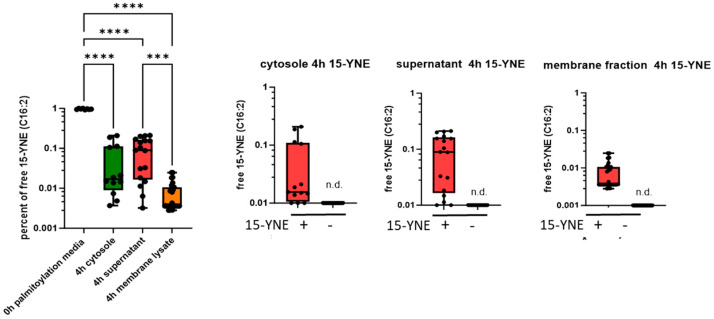
Ascertainment of free 15-YNE in different cell fractions by LC-HRMS. HCT-116 cells were treated for 4 h with or without 25 µM 15-YNE. Free 15-YNE was determined in palmitoylation media directly after application of 15-YNE (0 h) as well as after a 4 h incubation time (supernatant). Additionally, free 15-YNE was determined in the cytosol and membrane fraction of HCT-116 cells. The relative amount of free 15-YNE was calculated in relation to the level found in the supernatant (0 h). Statistical significance was calculated with one-way ANOVA with a mixed-effects analysis and a post- hoc Tukey’s multiple comparison test, α = 0.05. *** *p* < 0.001; **** *p* < 0.0001. n.d.: not detected. The data are presented as interleaved box and whiskers, Min. to Max., showing all points.

The free 15-YNE concentration in freshly prepared palmitoylation media (0 h) was set to 1 (100%), and the amount of free 15-YNE in cell culture media after a 4 h incubation time, as well as free 15-YNE in cell membrane fractions and cytosol, was related to free 15-YNE at 0 h (Figure 4). After 4 h of incubation, approximately 10% (mean, 9.6%) of the free 15-YNE (0 h) could be detected in the media. After 4 h of incubation time, approximately 6% (mean, 5.9%) of the free 15-YNE was detected in the cytosol and 0.8% (mean) was detected in the membrane fraction of HCT-116 cells. Notably, no 15-YNE (FA16:2) was detected in untreated cells, indicating that FA16:2 is not a naturally occurring lipid in our cells and that there is also no derivative with the same formula.

We also ascertained the relative amounts of other free fatty acids (16:1 palmitoleic acid and 16:0 palmitic acid) in these samples. However, all levels were below the detection limit (n.d.), with the exception of free 16:1 palmitoleic acid in cytosol and membrane lysates in non-15-YNE-treated cells (Supplement S3). C16:1 palmitoleic acid was also the only lipid that was detected in dialyzed FCS (Supplement S3). C16:1n7 palmitoleic acid is a known lipid hormone that plays a role in regulating systemic metabolism, including insulin metabolism [23,24]. It is produced in adipose tissue by the triglyceride lipase (Atgl) and increases after exercise [25]. Dialyzed fetal calf serum has a cut-off of 10 kDa; therefore, palmitoleic acid could still be detected. These data indicate that 15-YNE is clearly detectable and distinguishable from other C16 fatty acid derivatives and can be detected after 15-YNE treatment in all cell fractions as C16:2.

### 2.4. Effect of 15-YNE on the Cellular Lipidome

**Figure 5:**
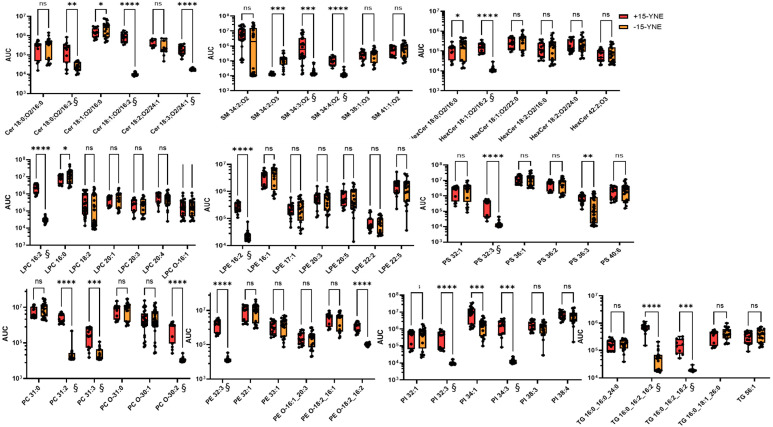
Detection of 15-YNE incorporation in different lipid classes. The lipidome of HCT-116 cells was analyzed with LC-HRMS after treatment of cells with or without 25 µM 15-YNE for 4 h. 15-YNE (isomer to FA16:2) incorporates into nearly all lipid classes and competes thereby with the endogenous C16 fatty acid. Relative quantification was based on chromatographic peak areas (AUC). Statistical analysis was performed by the mixed-effects model (REML) and a Turkey’s multiple comparisons test. *α* = 0.05; ns = not significant; * *p* < 0.05; ** *p* < 0.01; *** *p* < 0.001; **** *p* < 0.0001. The data are presented as interleaved box and whiskers, Min. to Max., showing all points. § For 15-YNE-containing lipids in −15-YNE-treated or clicked samples, no signals could be detected. To perform statistical analysis of these samples, the levels were filled by spectrum noise, calculated by the fill gap algorithm of the Compound Discoverer software (between an AUC of 104–105).

As treatment of cells with palmitic acid has been shown to alter the lipid composition in cells [26], we analyzed the lipidome in 15-YNE-treated cells using LC-HRMS, identifying 339 lipid species from 19 different lipid classes. Lipids were extracted from cells that were treated for 4 h with or without 25 µM 15-YNE. In the organic phase of membrane lysates, we could detect C16:2 in ceramides, hexosylceramides, and sphingomyelins, as well as in different phospholipids, such as lysophosphatidylcholines and lysophosphatidylethanolamines, and in triglycerides (Figure 5).

15-YNE is used for lipid metabolism in HCT-116 cells and incorporates into different lipid classes, which can be traced using LC-HRMS. The LC-HRMS analysis also shows that the naturally occurring lipid (C16:0) slightly decreases in 15-YNE-treated cells, indicating that 15-YNE supplants the naturally occurring lipid to a certain extent. How much 15-YNE is incorporated in different lipid species in comparison to the naturally occurring lipid is shown in Supplement S4. Calculating the ratio between the 15-YNE derivate and the natural lipid showed that 15-YNE is highly abundant after treatment of cells for 4 h in phosphatidic acid (PA), which is a central molecule in lipid metabolism and can be converted to triacylglyceride (TG), phosphatidylglycerol (PG), phosphatidylcholine (PC), phosphatidylethanol (PE), and phosphatidylinositol (PI). To investigate whether 15-YNE treatment affects the lipid equilibrium in our cells, we subjected the LC-HRMS data to LINEX analysis. We compared HCT-116 cells that were either treated with 15-YNE or not treated with 15-YNE.

**Figure 6:**
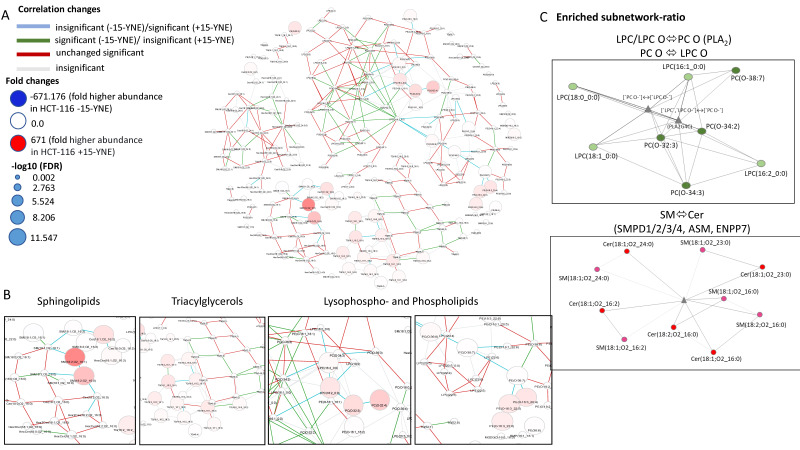
Network analysis of lipidomic data using LINEX2. (**A**) Depiction of LC-HRMS lipidomic data from HCT-116 cells treated with and without 15-YNE as a lipid network. The node size is scaled by the negative log10 of *p*-values for comparison between −15-YNE- and +15-YNE-treated cells. Lipids are colored according to their log fold change between HCT-116 −15-YNE- and HCT-116 +15-YNE cells. Blue indicates increased levels of lipids in the HCT-116 −15-YNE condition compared with the HCT-116 +15-YNE cells, and red indicates higher levels of lipids in the +15-YNE vs. −15-YNE constitution. The edges are colored by changes in the correlation for lipids from −15-YNE to +15-YNE-treated cells, with blue indicating a non-statistically significant correlation in the −15-YNE condition and a statistically significant correlation in the +15-YNE-treated cells, where the correlation has the same statistical sign. Green edge correlation changes indicate a statistically significant correlation in the −15-YNE condition and a statistically non-significant correlation in the +15-YNE-treated cells (same statistical sign). (**B**) Subnetwork showing Cer and SM, TG species, PC O-, LPC, LPC O-, PE O-, and LPE lipid nodes. (**C**) Enriched subnetworks generated by LINEX2. Spherical nodes depict lipid species, while triangular signs represent reaction types. The upper enriched subnetwork ratio shows two reaction triangulars, representing fatty acid transfer between PC O- and LPC O- lipids by the action of PLA2. The enriched subnetwork ratio in the bottom diagram depicts one triangular sign, showcasing lipid transfer between Cer and SM lipid species by the action of SMPD1–4 and SGMS.

As previously outlined, LINEX is an open-source web application designed for the analysis of lipidomic data, with the capacity to present the findings in the form of lipid networks. The computed data revealed no global changes in the lipidome but did reveal alterations in individual lipid species. As illustrated in Figure 6A,B, the network demonstrates statistically significant lipid changes within all lipid classes (sphingolipids, PE, PC LPC, and TG) (light pink to red nodes) between the two conditions (+15-YNE and −15-YNE). The most notable changes are related to the incorporation of C16:2 into the different lipid species in +15-YNE-treated cells compared with non-15-YNE-treated cells. Statistically significant reaction correlations were imaged via the network: The most prevalent correlations are represented by red edges, indicating a statistically significant correlation in −15-YNE and +15-YNE-treated cells, where the correlation has the same statistical sign. Additionally, blue edges represent a non-statistically significant correlation in the −15-YNE condition and a statistically significant correlation in the +15-YNE-treated cells. In addition, green edges correspond to a statistically significant correlation in the −15-YNE condition and a statistically non-significant correlation in the +15-YNE-treated cells. The majority of these correlation changes (green and blue edges) are associated with the incorporation of 15-YNE into lipids, leading to the formation of “new” lipids in the +15-YNE-treated cells. High fold changes with a positive statistical sign could therefore be observed in poly-unsaturated sphingomyelin (SM) (18:2;O2/16:1), SM (18:2;O2/16:0), Cer (18:1;O2/16:2), LPE (16:2), LPC (16:2), PC (O-32:3), and PC (O-32:4). Interestingly, the network (Figure 6A) shows a set of highly unsaturated ether-linked phosphatidylcholine (PC O) and ether phosphatidylethanolamine (PE O) species, connected by a blue reaction correlation, sharing identical FA signatures and converted into each other via headgroup modifications of PC (O-36:6), PC (O-38:7), PE (O38:7), PE (O-16:3_20:4), PE (O-16:3_22:5), and PE (O-16:3_22:6). The red nodes of a high circular magnitude (large fold changes) of these species in +15-YNE-treated HCT-116 cells indicate that these lipids succumb to a high turnover rate in HCT-116 cells as they are already traceable 4 h after 15-YNE treatment. Furthermore, our data show that 15-YNE is incorporated into sphingolipids as a second acyl chain (the reaction is catalyzed by CerS5 or CerS6) as well as after elongation to C18:2 as a sphingoid base that is desaturated to C18:3 (the light red node of a high circular magnitude for Cer (18:3;O2_24:1)). LINEX2 calculated an enriched subnetwork ratio as well (see Figure 6C). The enzymes that are probably involved in these metabolic changes are shown in gray triangles. These data also display a high turnover rate for LPC/LPC O and PC O, which is likely due to the high activity of phospholipase A2 (PLA2) enzymes (Figure 6C, upper image). The lower image of Figure 6C depicts the connection in the sphingolipid pathway that was identified with LINEX2, showing the metabolic correlations between sphingomyelin and ceramides that are performed by the activity of sphingomyelin phosphodiesterases (SMPD1–4), also known as acid- or neutral-sphingomyelinase (ASMase = SMPD1, NSMases = SMPD2–4) or sphingomyelin synthases.

In summary, our lipidomic data show that, after treatment of HCT-116 cells with 25 µM 15-YNE for 4 h, 15-YNE is incorporated into most of the lipid classes in these cells. A high abundance was detected in SM and PA, as well as metabolic derivatives of PA. Treatment with 15-YNE did not result in a significant global shift in lipid composition. Consequently, we conclude that 15-YNE is a suitable tracer for protein S-palmitoylation as well as lipid changes without affecting global lipid metabolism.

## 3. Discussion

Protein palmitoylation, a prevalent form of protein modification, exerts a significant influence on various cellular processes, including protein localization, activity, interaction, and degradation [27]. Consequently, it is unsurprising that protein palmitoylation is implicated in a multitude of cellular processes. For instance, S-palmitoylation of junctional adhesion molecules plays a regulatory role in cell–cell contacts and cell migration [28]. Additionally, cellular metabolism is regulated by palmitoylation of transport proteins such as fatty acid transporters (CD36) and glucose transporters (Glut1/4) [18,29,30]. It is noteworthy that deregulation of cell adhesion and metabolism is frequently observed in cancer cells. Consequently, the investigation of S-palmitoylation in cancer cells in diverse conditions and cancer stages holds promise in elucidating tumor-promoting mechanisms that are contingent on S-palmitoylation in cancer cells and may present novel treatment options for tumor therapies. To study this protein modification, we demonstrated the utility of metabolic labeling of cells with an alkyne-modified fatty acid and subsequent detection of palmitoylation using click chemistry. The findings reveal that the detection of protein S-palmitoylation using a clickable fluorescence molecule or biotin is both effective and beneficial for the study of protein palmitoylation. However, it is imperative to note that a meticulous separation of proteins from lipids is a prerequisite to minimize background signals. Concentration and time-dependent labeling experiments with 15-YNE demonstrate the efficacy of this method in detecting dynamic changes in protein palmitoylation within cells, which could not be investigated using ABE or acyl-RAC [5,6]. Furthermore, metabolic labeling with alkyne-modified fatty acids of varying chain length (e.g., C16:0, C18:0, C18:1) may also serve as a useful approach to study the substrate specificity of different zDHHCs and to determine the effect of protein lipidation with different fatty acids on protein function [31].

It has been demonstrated that fatty acid alkynes and fatty acid azides are utilized by acyl-CoA synthetases and zDHHCs in a comparable manner to naturally occurring lipids [11]. Changes in the expression level or activity of various DHHCs, as well as thioesterases, can influence protein palmitoylation and can be detected by this method, but it provides no information about the underlying mechanism. The observed discrepancies in protein palmitoylation between HCT-116 and Caco-2 cells may be associated with the expression level of the DHHC palmitoyl acyl transferase 6 (ZDHHC6), which is responsible for the palmitoylation of calnexin and is two-fold more highly expressed in HCT-116 cells compared with Caco-2 cells [18]. However, palmitoyl protein thioesterase 1 (PPT1), which cleaves palmitate from proteins and is 4-fold more highly expressed in Caco-2 cells than in HCT-116, could also be responsible for this discrepancy (The Human Protein Atlas [32]). The rapid decrease in calnexin S-palmitoylation after 6–16 h in Caco-2 and HCT-116 cells may be related to the fact that the half-life of non-palmitoylated calnexin is about 5 h whereas the half-life of palmitoylated calnexin is about 8 h [33]. The half-life of S-palmitoylation of calnexin is also approximately 8 h [33]. Although a pulse-chase experiment was not performed, it is not recommended to incubate cells with 15-YNE for more than 4 h because only 10% of free 15-YNE was detected in the cell culture medium after 4 h, which is insufficient for sufficient labeling of proteins.

Our second inquiry pertained to the impact of cell labeling with 15-YNE on lipid metabolism within cells. The detection of 15-YNE in nearly all lipid species suggests that it is utilized for lipid metabolism in a manner analogous to 16:0 palmitic acid. This has also been shown for fatty acid azide probes [11]. Alkyne fatty acids have been demonstrated to serve as effective tracers in the study of fatty acid beta-oxidation and metabolism [12,34,35]. These data suggest that these alkyne lipids are metabolized in a manner analogous to natural lipids. However, our observations revealed no significant alterations in the overall lipid composition of cells following treatment with 15-YNE. Consequently, fatty acid alkynes may also serve as viable tracers to monitor dynamic lipid changes in cells.

## 4. Study Limitations

The detection of protein lipidation by the method described here is limited to the visualization of reversible palmitoylation at cysteine residues of proteins, called S-palmitoylation, as the most common form. This method does not distinguish between other post-translational lipid palmitoylations such as N, O palmitoylation. However, N-, O-palmitoylation is relatively rare and irreversible and could not be cleaved by hydroxylamine; therefore, it is detected as a background signal. N-terminal myristoylation, S-prenylation, S-stearoylation, and others were not detected by this method [36,37,38,39,40]. In addition, each protein has a different half-life, which in turn requires prior optimization of the concentration and incubation times for adequate labeling with 15-YNE. As the 23 variants of the ZDHHC family of palmitoy-acyl-tranferase (PAT) enzymes have different FA specificities, besides palmitoylating only single-target proteins, it is recommended to test several FA species, such as C14, C16, and C18. It has been shown that cysteine acetylation with C16 or C18 fatty acids depends on the intracellular amount of the available acyl-CoA, which must be taken into account if acyl chain length is important [31]. As hydroxylamine-treated samples can be used as an alternative negative control in the S-palmitoylation quantification formula, the circumstance of protein degradation at high hydroxylamine concentrations has to be considered.

The applied LC-HRMS method separates lipids based on the mass-to-charge ratio and chromatographic retention using reversed-phase chromatography. The aim is to cover as many lipid classes as possible in a short run; therefore, isomers with differing fatty acid compositions are not separated. Furthermore, 15-YNE is isomeric to FA16:2 and therefore cannot be distinguished. Investigating the data-dependent MS/MS fragmentation patterns of ceramides and TG in positive ion mode, or of phospholipids in negative ion mode, provides insights into the qualitative fatty acid composition based on the exact mass of the fatty acid. However, in the case of coeluting isomers, it cannot be considered quantitative, two double bonds and a triple bond cannot be distinguished, and the double or triple bond positions are unknown. Therefore, experiments should always include suitable controls for the relative quantification of the modified lipids in comparison to endogenously present isomers. Furthermore, the ionization efficacy of lipids containing 15-YNE is likely different from that of palmitic-acid-containing lipids, allowing only for relative quantification in the absence of suitable reference standards.

## 5. Materials and Methods

### 5.1. Cell Culture

The human colon cancer cell lines Caco-2 (ATCC^®^ HTB-37™) and HCT116 (ATCC^®^ CCL-247™) were procured from the American Type Culture Collection (ATCC, Manassas, VA, USA). The HCT-116 and Caco-2 cells were transformed with a lentivirus containing a non-coding short hairpin RNA (shRNA), as previously described in [41]. Caco-2 cells were cultivated in Gibco^®^ Minimum Essential Medium (MEM) containing Earle’s Salts and L-glutamine (Thermo Fisher Scientific, Waltham, MA, USA), with the medium being enriched by the addition of 10% (*v*/*v*) fetal calf serum (FCS), 1% (*v*/*v*) Gibco^®^ MEM Non-Essential Amino Acids (NEAA; Thermo Fisher Scientific), and 1% (*v*/*v*) penicillin (50 U/mL)/streptomycin (50 µg/mL). The HCT-116 cell lines were cultivated in McCoy’s 5A medium (ATCC^®^ 30-2007™), with the addition of 10% (*v*/*v*) fetal calf serum (/bovine) to the medium and 1% (*v*/*v*) penicillin (50 U/mL)/streptomycin (50 µg/mL) (both from Gibco^®^, Thermo Fisher Scientific, Waltham, MA, USA). Both cell lines were cultured at 37 °C in an atmosphere containing 5% CO_2_.

### 5.2. Detection of Protein S-Palmitoylation via Click Chemistry

S-Palmitoylation of proteins is detected by labeling cells with 15-YNE, which is subsequently detected either by Cy5.5-azide or Picolyl-azide-PEG4-biotin (biotin-azide) coupled to 15-YNE via click chemistry. In brief, HCT-116 or Caco-2 cells were incubated with 10, 15, or 25 µM 15-YNE for up to 16 h (to determine time and concentration dependency). Afterwards, cells were harvested, and membranes were separated by centrifugation (100,000× *g*, 4 °C, 1 h). Protein concentrations in membrane fractions were determined by the Pierce BCA assay according to the manufacturer’s instructions (Thermo Fisher Scientific, Dreieich, Germany). For the click reaction, 50 µg and 400 µg of protein were used with either Cy5.5-azide or biotin-azide, respectively. The click reaction was initiated by the addition of CuSO_4_ (Sigma Aldrich, Merck, Darmstadt, Germany), Tris[(1-benzyl-1*H*-1,2,3-triazol-4-yl)methyl]amine (TBTA, Sigma-Aldrich, Merck, Germany), Cy5.5-azide, or Picolyl-Azide-PEG4-Biotin (Jena Bioscience, Jena, Germany), along with at least Tris-(2-carboxyethyl)phosphine, hydrochloride (TCEP, Sigma-Aldrich, Merck, Germany). The click reaction was incubated for 1 h at 21 °C with gentle rocking on a shaker platform. After one hour, the reaction was stopped by adding EDTA (10 mM final concentration) (Thermo Fisher Scientific, Dreieich, Germany). A click reaction can be validated by treating samples after the click chemistry reaction with 0.6 M hydroxylamine (Sigma Aldrich, Merck Germany), a reagent that reduces the thioester bond between the palmitoyl moiety and sulfhydryl groups, thereby removing the 15-YNE clicked to Cy5.5- or biotin-azide from proteins. Membrane proteins were separated from lipids by methyl-tert-butyl-ether (MTBE, Sigma Aldrich Merck, Germany)/methanol extraction. Proteins clicked to Cy5.5-azide were loaded onto an SDS-polyacrylamide gel and transferred to a nitrocellulose membrane. Subsequently, the membrane was blocked with 5% bovine serum albumin (Sigma Aldrich Merck, Germany) and incubated with an anti-calnexin antibody or anti-SDHA antibody and secondary antibodies coupled to IRdye800. Cy5.5 (700 nM) and the secondary antibodies (800 nm) were detected by a LICOR Odyssey CLX imaging system (LICORbio, Bad Homburg, Germany).

The isolation of biotinylated proteins from cell lysates was achieved through the use of 200 μL of clicked, MTBE/methanol-treated protein extracts, which were then incubated with 20 μL of Dynabeads™ MyOne™ Streptavidin C1. The samples were then subjected to gentle mixing by means of pipetting up and down, followed by an overnight incubation with rotation at 4 °C. Subsequent to this, the beads were thoroughly washed, and S-acetylated proteins were eluted. The eluate was subsequently loaded onto an SDS-polyacrylamide gel and transferred to a nitrocellulose membrane. Subsequent steps were performed as previously outlined.

### 5.3. Non-Targeted LC-HRMS Analysis of Lipids

LC-HRMS analysis of lipids was conducted as previously described [42]. A total of 50 µL of the sample was mixed with 75 µL of internal standards in methanol, 250 µL of MTBE, and 10 µL of 50 mM ammonium formate solution. This was followed by vortexing for 1 min and centrifugation (5 min, 18,213× *g*, 4 °C). After transferring the upper organic layer, 100 µL of an MTBE/methanol/water solution (10:3:2.5, *v*/*v*/*v*) was added, followed by vortexing and centrifugation. The organic layers were combined, evaporated under nitrogen at 45 °C for 10 min, and stored at <−70 °C. Before analysis, samples were dissolved in 100 µL of methanol. Data were acquired in both positive and negative ion mode on an Orbitrap Exploris 480 with a Vanquish Horizon UHPLC system using XCalibur v4.4 software (all Thermo Fisher Scientific, Dreieich, Germany). The mass resolving power was set at 120,000 (MS1) and 15,000 (data-dependent MS2). The LC method involved a gradient elution using a Zorbax RRHD Eclipse Plus C8 column (Agilent, Waldbronn, Germany) (2.1 × 50 mm, 1.8 µm, identical guard column) with mobile phase A (water with 10 mM ammonium formate and 0.1% formic acid) and mobile phase B (acetonitrile/isopropanol 2:3 *v*/*v* with 0.1% formic acid).

The evaluation of raw data in the positive ion mode was performed using Compound Discoverer software version 3.3 (Thermo Fisher Scientific). To manage the substantial volume of data, the dataset was partitioned into five segments, with each segment comprising approximately 100 samples and more than 5000 aligned features. For the purpose of feature reduction, a two-sample *t*-test was conducted for the first batch, which was considered a representative sample. This test was performed for all cell lines, comparing samples that received 15-YNE treatment with those that did not. Only features with a *p*-value (unadjusted) of less than 0.05 and log2 fold changes of less than −1 or more than 1 in at least one of the comparisons were retained. Furthermore, features lacking a molecular formula or MS/MS spectra, or for which the mass error exceeded 5 ppm, were removed. Afterwards, the five datasets were merged by aligning the *m*/*z* and retention time (RT) features using the Manhattan distance. Features that exhibited a mass error exceeding 5 ppm or an RT difference greater than 0.1 min compared with the reference batch were excluded from further analysis. The results were then normalized using probabilistic quotient normalization based on the complete dataset, without reduction to significantly altered features [43]. The methodology employed for the handling of data in R is provided in the Supplement S1. The relative quantification of 15-YNE, palmitic acid (FA16:0), and palmitoleic acid (FA16:1) was achieved by evaluating the data acquired in negative ion mode using TraceFinder v5.1 software (Thermo Fisher Scientific). The mass tolerance was set to 5 ppm based on the theoretical mass. A comprehensive description of all measurement and evaluation parameters can be found in Supplement S2.

### 5.4. Analysis of Lipidomic Data by LINEX2

In order to utilize the lipidomic data for LINEX2, we first converted lipid names from the lipidomic data with Lipid LynxX [44]. This allowed us to standardize lipid terms according to the utilized nomenclature of LINEX^2^ [16] for recognition of lipid names at the sum-species level or molecular-species level. In instances where lipid class information was not available, we undertook a manual extension of the data. LINEX^2^ processes these data and depicts a network of reactions/conversions that are shown as edges. The encoded reactions are based on customizable rules. Default settings comprise common reactions of glycero-, glycerophospho-, and sphingolipids as well as typical fatty acid modifications. The strongest matches of lipid metabolic/reaction networks were split into two categories: class- or headgroup-related transformations and fatty-acid-related transformations. Moreover, fatty-acid reactions were calculated with subsequent optional modifications (elongation, desaturation, and oxidation), linking lipids of the same class if they varied in a chain length of 2. The edges are connected by nodes that depict the lipid class reactions. At least 46 common lipid classes are supported. A hyper-network is calculated based on edges between a lipid class reaction node and all lipid species participating in this reaction. Each hyper-edge corresponds to a lipid species reaction (lipid substrates, lipid products, and lipid class reaction nodes). The network extension method is determined by universal reaction rules with an obligatory manual selection of distinct reaction databases. To exclude bias, the network extension prevents the participation and preference of conventionally studied enzymes for the calculated metabolic reactions by using lipid class reactions instead of enzymes. At present, the algorithm is limited in certain ways, such as depicting a set of candidate enzymes assigned to the same reaction type rather than highlighting individual enzymes. A potential dysregulation is quantified by the relative ratio-change of lipid substrates to lipid products or the difference between two experimental setups. Computing a potential dysregulation, the algorithm takes the reversibility of the substrate/product species into account. The reaction network is utilized to search for the most dysregulated subnetwork. The calculated dysregulation (enriched subnetwork) is computed via an empirical *p*-value estimation procedure.

### 5.5. Statistics

The data are presented as mean values ± standard error of the mean (SEM) or as a box and whiskers plot (min to max). Statistical comparisons were performed with one-way or two-way ANOVA with Dunnett’s, Sidák’s, or Tukey’s multiple comparison test, which was performed using GraphPad Prism version 9.3 for Windows (GraphPad Software, San Diego, CA, USA). When groups were compared with various n values, the mixed-effects model (REML) was used. For time courses, the area under the curve (AUC) values were calculated and analyzed by one-way ANOVA. Data from two groups were analyzed using unpaired t-tests. For all significant changes, the *p*-value was defined as follows: * = 0.05; ** = 0.01; *** = 0.001; **** = 0.0001.

To assert the biochemical association between lipid species and the lipidomic data of computed networks, LINEX implements statistical metrics. For the analysis of alterations in lipid levels between different experimental conditions, an unpaired parametric test (*t*-test), a non-parametric test (Wilcoxon rank-sum test or Mann–Whitney U rank test), and a paired parametric test (Wilcoxon signed-rank test) were provided. *p*-values are depicted as the false-discovery rate (FDR), corrected using the Benjamini–Hochberg procedure [45]. Fold changes were calculated via the SciPy package in python (SciPy 1.0) in conjunction with the stats model package, visualizing effect size.

## 6. Conclusions

In summary, the detection of protein palmitoylation through the use of fluorescent-azide or biotin-azide is equally effective following the labeling of cells with 15-YNE. Furthermore, the incorporation of fatty acid alkynes into lipids facilitates the monitoring of alterations in lipid metabolism via LC-HRMS. Given that protein palmitoylation and protein localization can be influenced by the lipid composition of membranes, the assessment of both in a single step constitutes a useful approach to investigating interrelationships.

## Data Availability

All original data will be made available by the corresponding author upon request.

## Supplementary Materials

The following supporting information can be downloaded at: www.mdpi.com/article/10.3390/molecules30020377/s1, Supplement S1 (PDF): *m*/*z* and RT feature alignment_manhatten-distance, Supplement S2 (Excel file): Lipidomic information for analyzing 15-YNE, Supplement S3 (Figure): 15_YNE in different cell fractions, Supplement S4 (Figure): Tracing of 15-YNE in different lipids.
