# Supplementary material for: Cell Labeling with 15-YNE Is Useful for Tracking Protein Palmitoylation and Metabolic Lipid Flux in the Same Sample
Source: Molecules. 2025 Jan 17;30(2):377. doi: 10.3390/molecules30020377 (PMC11767944; doi:10.3390/molecules30020377)
Supplement: Supplementary file 1 [file molecules-30-00377-s001.zip › Supplement 1 alignment_manhattan-distance.pdf]

m/z & RT feature alignment using manhattan distance

Samuel Rischke, Lisa Hahnefeld

19.07.2024

```r
library(dplyr)
library(readxl)
library(openxlsx)
library(purrr)
library(sqldf)
library(tibble)
```

Functions

```r
### Function to extract the desired part of the string
extract_name <- function(name, polarity = "pos") {
  # Use regular expression to match the desired pattern
  if(polarity == "pos"){
  sub(".*pos_(.*)\\.raw.*", "\\1", name)
  }else{sub(".*neg_(.*)\\.raw.*", "\\1", name)}
}
#explanation:
#.*pos_: Matches any character (.) zero or more times (*) until "pos_" is found.
#(.*): Captures any character (.) zero or more times (*) and stores it in a group (i.e., the part we wa
#\\.raw: Matches the literal ".raw".
#.*: Matches any character (.) zero or more times (*).
#\\1: Refers to the first captured group (i.e., the part of the string we want to keep).

### Normalisation factors and probabilistic quotient normalization

# Function to calculate normalization factors based on mean or median
# Can normalize relative to specified QC samples, otherwise relative to all samples
NormFac <- function(X, n = "median", QC = NULL) {
  X.NormFac <- rep(NA, ncol(X))
  if (!is.null(QC)) {
    if (length(QC) == 1) {
      X.NormFac <- as.numeric(X[QC, ])
    } else {
      if (n == "mean") {
        X.NormFac <- as.numeric(colMeans(X[QC, ]))
      } else if (n == "median") {
        X.NormFac <- as.numeric(apply(X[QC, ], 2, median))
      }
    }
```

```r
  } else {
    if (n == "mean") {
      X.NormFac <- as.numeric(colMeans(X))
    } else if (n == "median") {
      X.NormFac <- as.numeric(apply(X, 2, median))
    }
  }
  Y.NormFac <- rep(NA, nrow(X))
  for (a in seq(nrow(X))) {
    # normalization factor, sample results must be multiplied with this factor
    Y.NormFac[a] <- median(as.numeric(X.NormFac / X[a, ]))
    # normalization factor, sample result must be divided for normalization
    #Y.NormFac[a] <- median(as.numeric(X[a, ] / X.NormFac))
  }
  # Create a data frame to return, preserving row names from X
  result <- data.frame(Sample.Name = rownames(X), Normalisation.Factor = Y.NormFac)
  return(result)
}

# Function to calculate normalized values (e.g. peak area) based on mean or median
# Can normalize relative to specified QC samples, otherwise relative to all samples
pqn <- function(X, n = "median", QC = NULL) {
  X.norm <- matrix(nrow = nrow(X), ncol = ncol(X))
  colnames(X.norm) <- colnames(X)
  rownames(X.norm) <- rownames(X)

  if (!is.null(QC)) {
    # if QC vector exists, use this as reference spectrum
    if (length(QC) == 1) {
      # only 1 reference sample given
      mX <- as.numeric(X[QC, ])
    } else {
      if (n == "mean") {
        mX <- as.numeric(colMeans(X[QC, ]))
      }
      if (n == "median") {
        mX <- as.numeric(apply(X[QC, ], 2, median))
      }
    }
  } else {
    # otherwise use the mean or median of all samples as reference sample
    if (n == "mean") {
      mX <- as.numeric(colMeans(X))
    }
    if (n == "median") {
      mX <- as.numeric(apply(X, 2, median))
    }
  }

  # do the actual normalization
  for (a in 1:nrow(X)) {
    X.norm[a, ] <- as.numeric(X[a, ] / median(as.numeric(X[a, ] / mX)))
  }
```

```
  return(X.norm)
}
```

Prepare and load data (xlsx) into a list:

Datasets must contain columns called 'm/z' and 'RT [min]'. Datasets are Compound Discoverer Excel exports of the Compounds list. Before export, the columns "Area" and optionally "#Adducts", "Gap Fill Status" and "Peak Rating" should be enabled. All datasets should be merged into a single xlsx file with one table/sheet per dataset. The order of the sheets will be retained for the final dataset.

```
# path and file names
file_path = "Y:\\R_transfer"
#file_path = "F:\\00_Freitag+Montag\\Auswertung Nadine"
#file names with file extension (should be .xlsx)
file_name = "pos_112-03_CD_all_sets_all_features.xlsx"
num_sheets = 5
study_name = "pos_112-03"
current_date = format(Sys.Date(), "%Y-%m-%d")

# set tolerance limits
mz_tolerance <- 5 / 1e6 # m/z tolerance, usually 5 ppm
rt_tolerance <- 0.1 # retention time tolerance in minutes


# load data into a list
ls = lapply(seq(num_sheets),function(i){
  x = read_excel(paste(file_path, file_name, sep="\\"), sheet = i)
}) %>% suppressMessages() %>% suppressWarnings()

# set and remove reference dataset
ref = ls[[1]] %>% as.data.frame
ls = ls[-1]
```

Align Features

Calculate manhattan distance to the reference dataset (= first dataset). Distance is calculated based on m/z and RT values and the feature with the minimal distance is aligned.

```
#get reference m/z and RT values
ref_df = data.frame(mz = ref$`m/z`,
                    rt = ref$`RT [min]`)


#extract m/z and RT values from each list
#Compute Distances and Finding Minimums
ls_dist = lapply(seq_along(ls),function(i){
  x = data.frame(mz = ls[[i]]$`m/z`,
                 rt = ls[[i]]$`RT [min]`)
  x_dist = dist(rbind(ref_df,x),method = "manhattan")
  x_dist = as.matrix(x_dist)
  x_dist = x_dist[1:nrow(ref),-c(1:nrow(ref))]
```

```
  which.is.min = apply(x_dist,1,which.min)
  y = ls[[i]][which.is.min,] %>% as.data.frame
  return(y)
})

#recombine datasets
merged_data = append(list(ref),ls_dist)
dim.test = merged_data
# horizontally combine data frames
merged_data = do.call(cbind,merged_data)

# select all columns that contain "m/z" or "RT"
mz_rt_indices = grep(paste(c("m/z", "RT"), collapse = "|"), colnames(merged_data))
mz_indices = grep(paste(c("m/z"), collapse = "|"), colnames(merged_data))
rt_indices = grep(paste(c("RT"), collapse = "|"), colnames(merged_data))
mz_rt_data = merged_data[, c(mz_indices,rt_indices)]

head(mz_rt_data)
```

```
##        m/z    m/z.1    m/z.2    m/z.3    m/z.4 RT [min] RT [min].1 RT [min].2
## 1 531.2721 531.2721 531.2721 531.2724 531.2722    1.932      1.932      1.948
## 2 758.5682 758.5686 758.5682 758.5682 758.5683    4.323      4.327      4.316
## 3 758.5684 758.5686 758.5682 758.5682 758.5683    4.327      4.327      4.316
## 4 786.5997 786.5999 786.5998 786.5998 786.6000    4.780      4.777      4.793
## 5 531.2721 531.2721 531.2721 531.2724 531.2722    2.074      2.061      1.948
## 6 760.5839 760.5840 760.5841 760.5840 760.5841    4.691      4.698      4.706
##   RT [min].3 RT [min].4
## 1      1.929      1.929
## 2      4.304      4.305
## 3      4.304      4.305
## 4      4.799      4.789
## 5      1.929      2.060
## 6      4.726      4.711
```

Check feature alignment

Label features, which do not meet the alignment criteria. Tolerance is applied in relation to the first reference dataset.

```
df = mz_rt_data
#reference values
mz_ref = df[,1]
rt_ref = df[,(num_sheets + 1)]



# Create a logical matrix checking if each mz value is within the tolerance of the reference dataset
within_tolerance_mz <- sapply(1:num_sheets, function(i) {
  abs(df[, i] - mz_ref)/ df[,i] > mz_tolerance
})
within_tolerance_rt <- sapply((num_sheets+1):(num_sheets*2), function(i) {
  abs(df[, i] - rt_ref) > rt_tolerance
```

```r
})

# Convert logical matrix to data frame and set appropriate column names
remove_results <- as.data.frame(cbind(within_tolerance_mz | within_tolerance_rt))
names(remove_results) <- paste("dataset", 1:num_sheets, sep = "")

head(remove_results)
```

```
##   dataset1 dataset2 dataset3 dataset4 dataset5
## 1    FALSE    FALSE    FALSE    FALSE    FALSE
## 2    FALSE    FALSE    FALSE    FALSE    FALSE
## 3    FALSE    FALSE    FALSE    FALSE    FALSE
## 4    FALSE    FALSE    FALSE    FALSE    FALSE
## 5    FALSE    FALSE     TRUE     TRUE    FALSE
## 6    FALSE    FALSE    FALSE    FALSE    FALSE
```

Remove values of features not meeting alignment criteria

mass error > 5e-06 ppm. RT deviation > 0.1 min.

```r
#Print(paste("mass error > ",toString(mz_tolerance), " ppm",sep = ""))
#Print(paste("RT deviation > ", toString(rt_tolerance, " min", sep=""))
#column numbers of list data
nc = sapply(dim.test,ncol)

#create a a list of logic matrices, repeating the values to remove for each column in the respective da
eval_mat = lapply(seq(num_sheets),function(i){
  x = matrix(rep(remove_results[,i],nc[i]),nrow = nrow(merged_data),byrow = F)
  return(as.data.frame(x))
})
eval = do.call(cbind,eval_mat)
names(eval) = paste("V",1:ncol(eval),sep="")
#clean data (replace all values with True with N/A)
# eval must be a proper logical matrix
merged_data_cleaned = merged_data
merged_data_cleaned[as.matrix(eval)] = NA
```

Split sample specific results from compound identification

Splits the data into two tables: one for further statistical analysis, only containing minor information about the features, and one to check the feature annotation. The feature list can be reduced at this step or in the beginning, by selecting a subset in Compound Discoverer software. Duplicate samples (e.g. controls which were reevaluated in multiple batches) are retained only from the first appearance, but can be manually checked. Gap Fill Status (required to check true number of missing values) currently not used.

```r
### Sample specific results
# select column (partial) names to be kept for sample results
sample_col_names = c("Name","m/z","RT","Area: ")
sample_col_indices = grep(paste(sample_col_names, collapse = "|"), colnames(merged_data_cleaned))
sample_results = merged_data_cleaned[,sample_col_indices]
remove_col_indices = grep("Group Area:",colnames(sample_results))
```

```
sample_results = sample_results[,-remove_col_indices]

# clean column names
# check function, if sample naming from Compound Discoverer does not follow "pos_SampleName.raw (F Samp
colnames(sample_results) = sapply(colnames(sample_results), extract_name)

# check for duplicate columns/samples
duplicates <- colnames(sample_results)[duplicated(colnames(sample_results))]
cat("The following columns apear more than once:\n")
```

```
## The following columns apear more than once:
```

```
print(duplicates)
```

```
##  [1] "112-03_Blank"         "112-03_zero"          "112-03_KP_20230222_7"
##  [4] "112-03_KP_20230222_8" "112-03_Blank"         "112-03_zero"
##  [7] "112-03_KP_20230222_7" "112-03_KP_20230222_8" "112-03_Blank"
## [10] "112-03_zero"          "112-03_KP_20230222_7" "112-03_KP_20230222_8"
## [13] "112-03_Blank"         "112-03_zero"          "112-03_KP_20230222_7"
## [16] "112-03_KP_20230222_8"
```

```
# select subset of duplicate columns for manual checking
duplicate_samples = sample_results[,grep(paste(duplicates, collapse = "|"), colnames(sample_results))]
#remove duplicate Name, m/z and RT values
duplicate_mz = c("Name\\.", "m/z\\.", "RT \\[min\\]\\.")
remove_col_indices = grep(paste(duplicate_mz,collapse = "|"),colnames(sample_results))
sample_results = sample_results[,-remove_col_indices]

# set unique names
colnames(duplicate_samples) = make.names(colnames(duplicate_samples), unique = T)
# order columns alphabetically
duplicate_samples = duplicate_samples[, order(colnames(duplicate_samples))]

# keep only duplicate columns from reference dataset (= first appearance)
sample_results = sample_results[, !duplicated(colnames(sample_results))]
# new identifier column
sample_results = cbind(feature = paste(sample_results$`m/z`, sample_results$`RT [min]`, sep = "@"), samp


### Compound identification specific results
# select columns required for compound identification
feature_col_names = c("Name","Formula","Annot.","Calc. MW","m/z","RT","Area (Max.)","mzVault Results","
feature_col_indices = grep(paste(feature_col_names, collapse = "|"), colnames(merged_data_cleaned))
# select feature columns
feature_results = merged_data_cleaned[,feature_col_indices]
# set unique names
colnames(feature_results) = make.names(colnames(feature_results), unique = T)
# order columns alphabetically
feature_results = feature_results[,order(colnames(feature_results))]
# new identifier column
feature_results = cbind(feature = paste(feature_results$m.z,feature_results$RT..min., sep = "@"), featu
```

Normalize sample results using Probabilistic quotient normalization

```
# normalize results on qc samples?
use_qcs = F
# Partial string for recognition of qc samples
qc.ident = "QC-G"

# use inclusion list to select samples to normalize, if false use exclusion of sample groups
use_include_sample = T
inclusion_list_name = "2024-07-01_sample_inclusion_list.xlsx"
# partial string(s) for sample names to be removed
#rem_sample_names = c("Blank", "KP", "zero")
# Partial string(s) for sample groups to be removed
rem_sample_groups = c("blank","plasma","QC", "media","FCS")
file_group_name = "2024-07-01_sample-groups.xlsx"

# get dimensions
analytes<-unique(sample_results$feature)
samples_raw<-unique(colnames(sample_results)[5:ncol(sample_results)])
# remove unnecessary columns (for PQN)
mat = sample_results
rownames(mat) = mat$feature
mat = mat[,-c(1:4)]

#apply sample inclusion or exclusion by sample group
if(use_include_sample){
  # load samples to include
  inclusion_list = read_xlsx(paste(file_path,inclusion_list_name, sep = "\\"))
  included_samples = inclusion_list$`File Name`
  # filter samples to include
  mat = mat[,included_samples, drop = F]
  cat("Samples for PQN normalization were selected by inclusion list.", "\n")
}else{
  # load group names
  sample_groups = read_xlsx(paste(file_path,file_group_name, sep = "\\"))
  # filter samples + groups not required for normalization
  rem_group_ind = grep(paste(rem_sample_groups, collapse = "|"), sample_groups$Group)
  rem_samples = sample_groups[rem_group_ind,1]
  rem_samples_ind = grep(paste(rem_samples$`File Name`,collapse = "|"), colnames(mat))
  mat = mat[,- rem_samples_ind]
  cat("Samples for PQN normalization were selected by excluding sample groups.", "\n")
}
```

```
## Samples for PQN normalization were selected by inclusion list.
```

```
#transpose data frame, samples should be in rows and features in columns
mat = t(mat)
samples = rownames(mat)
mat = apply(mat,2,as.numeric)
rownames(mat) = samples

#get row index of QC samples
```

```
if(use_qcs){qc<-grep(c(qc.ident),samples)}

# check missing values
mat[mat == 0] <- NA
all.na<-colSums(is.na(mat))==nrow(mat)
cat(c("Values for",sum(all.na),"features are missing completely."), "\n")
```

```
## Values for 0 features are missing completely.
```

```
#remove all features with > 10% missing values
ten.na<-colSums(is.na(mat))/nrow(mat)>0.1
cat(c("Values for",sum(ten.na),"features are missing in more than 10%."), "\n")
```

```
## Values for 950 features are missing in more than 10%.
```

```
mat<-mat[,!ten.na]

# replace remaining missing values with min/2
#in some cases there are no more missing values (due to Fill gap function in CD)
isna <- which(is.na(mat), arr.ind = T)
if(length(isna) >0){
  minhalf <- t(as.data.frame(apply(mat, 2, function(x) min(x, na.rm = T))) / 2)
  minhalf <- minhalf[rep(seq_len(nrow(minhalf)), each = nrow(mat)), ]
  mat[isna] <- minhalf[isna]
  }else{
  cat("No missing values needed replacement with min/2", "\n")
}
```

```
## No missing values needed replacement with min/2
```

```
# collect all removed features
rem_analytes<-c(names(all.na[all.na==TRUE]),
        names(ten.na[ten.na==TRUE]))
cat(length(rem_analytes),"features have been omitted in total.", "\n")
```

```
## 950 features have been omitted in total.
```

```
# apply PQN
# At the beginning of the script decide whether pqn.mat = pqn.all (False) or pqn.mat = pqn.qc (True)
cat("Normalization was performed adjusting to QC values: ", use_qcs)
```

```
## Normalization was performed adjusting to QC values:  FALSE
```

```
if (use_qcs) {
  norm.fac <- NormFac(mat, n = "median", QC = qc)
  pqn.qc <- pqn(mat, n = "median", QC = qc)
  pqn.all <- pqn(mat, n = "median", QC = NULL)
  cat("The QCs were identified by: ", qc.ident, "\n", "There are differences in normalization due to QCs
  pqn.mat = pqn.qc
```

```
} else {
  norm.fac <- NormFac(mat, n = "median", QC = NULL)
  pqn.mat = pqn(mat, n = "median", QC = NULL)
}
```

The normalized data must be brought back into the original form. This means that they are, for example, brought back together with information about the peak shape. Specifically, analytes and samples excluded from normalization are added back so that a "sparse matrix" is created. This is linearized into a vector of normalized areas (and missing values). This vector becomes the "PQN Area" column in the output result report. In some case returning the normalization factors per sample is sufficient.

```
# add non-normalized samples to normalization factors
all.normfac = data.frame(Sample.Name = samples_raw)
# add sample groups (from inclusion or group exclusion list)
if(use_include_sample){
  sample_types = select(inclusion_list, `File Name`, `Group`)
}else{
sample_types = select(sample_groups, `File Name`, `Group`)
}
colnames(sample_types) <- c("Sample.Name", "Group")
all.normfac <- all.normfac %>%
  left_join(sample_types, by = "Sample.Name") %>%
  distinct() # ensure no duplicate rows
all.normfac <- left_join(all.normfac, norm.fac, by = c("Sample.Name"))
all.normfac[is.na(all.normfac)] <- 1 # replace missing values with 1

## reformat normalized peak areas
# matrix is so large that analytes must be in rows not columns
pqn_wide <- t(pqn.mat)

# add compound infos
pqn_wide = cbind(pqn_wide, feature = rownames(pqn_wide))
sample_results_PQN_allFeat =sample_results[,c(1:4)] %>%
  left_join(pqn_wide, by = "feature", copy = T) %>%
  distinct() #ensure no duplicate rows

# filter out removed compounds
sample_results_PQN = subset(sample_results_PQN_allFeat, !(feature %in% rem_analytes))
feature_results_PQN = subset(feature_results, !(feature %in% rem_analytes))
```

Check for remarkable normalization factors.

```
limit_high = 1.5
limit_low = 0.5

norm.high = norm.fac$Sample.Name[norm.fac$Normalisation.Factor > limit_high]
norm.low = norm.fac$Sample.Name[norm.fac$Normalisation.Factor < limit_low]

cat("Normalization factors > ", limit_high, ": ", norm.high, "\n")
```

```
## Normalization factors >  1.5 :
```

```r
cat("Normalization factors < ", limit_low, ": ", norm.low, "\n")
```

```
## Normalization factors <  0.5 :  112-03-002
```

Save combined datasets

```r
# readxl library not so well suited for writing xlsx files, switch to openxlsx (libraries should not in
# List of data frames to save and corresponding sheet names
data_to_save <- list(normalization_factors = all.normfac,
                     sample_results_PQN = sample_results_PQN,
                     feature_results_PQN = feature_results_PQN,
                     #sample_results_PQN_allFeat = sample_results_PQN_allFeat,
                     #feature_results_all = feature_results,
                     #removed_samples_PQN = rem_samples #only if samples selected by exclusion
                     removed_analytes_PQN = rem_analytes,
                     duplicate_samples = duplicate_samples,
                     sample_results_unadjusted = sample_results,
                     #merged_data_Cleaned = merged_data_cleaned,
                     #merged_data_uncleaned = merged_data,
                     mz_rt_uncleaned = mz_rt_data)

# Create a new workbook
wb <- createWorkbook()

# Add worksheets and write data frames in one line each using a loop
for (sheet_name in names(data_to_save)) {
  addWorksheet(wb, sheet_name)
  writeData(wb, sheet = sheet_name, data_to_save[[sheet_name]])
}

# Save the workbook to a file
saveWorkbook(wb, paste(file_path,"\\",current_date,"_",study_name,"_results_full.xlsx", sep = ""), overw
```
