# Supplementary figures and images for: Cell Labeling with 15-YNE Is Useful for Tracking Protein Palmitoylation and Metabolic Lipid Flux in the Same Sample
Source: Molecules. 2025 Jan 17;30(2):377. doi: 10.3390/molecules30020377 (PMC11767944; doi:10.3390/molecules30020377)

## Slide 1
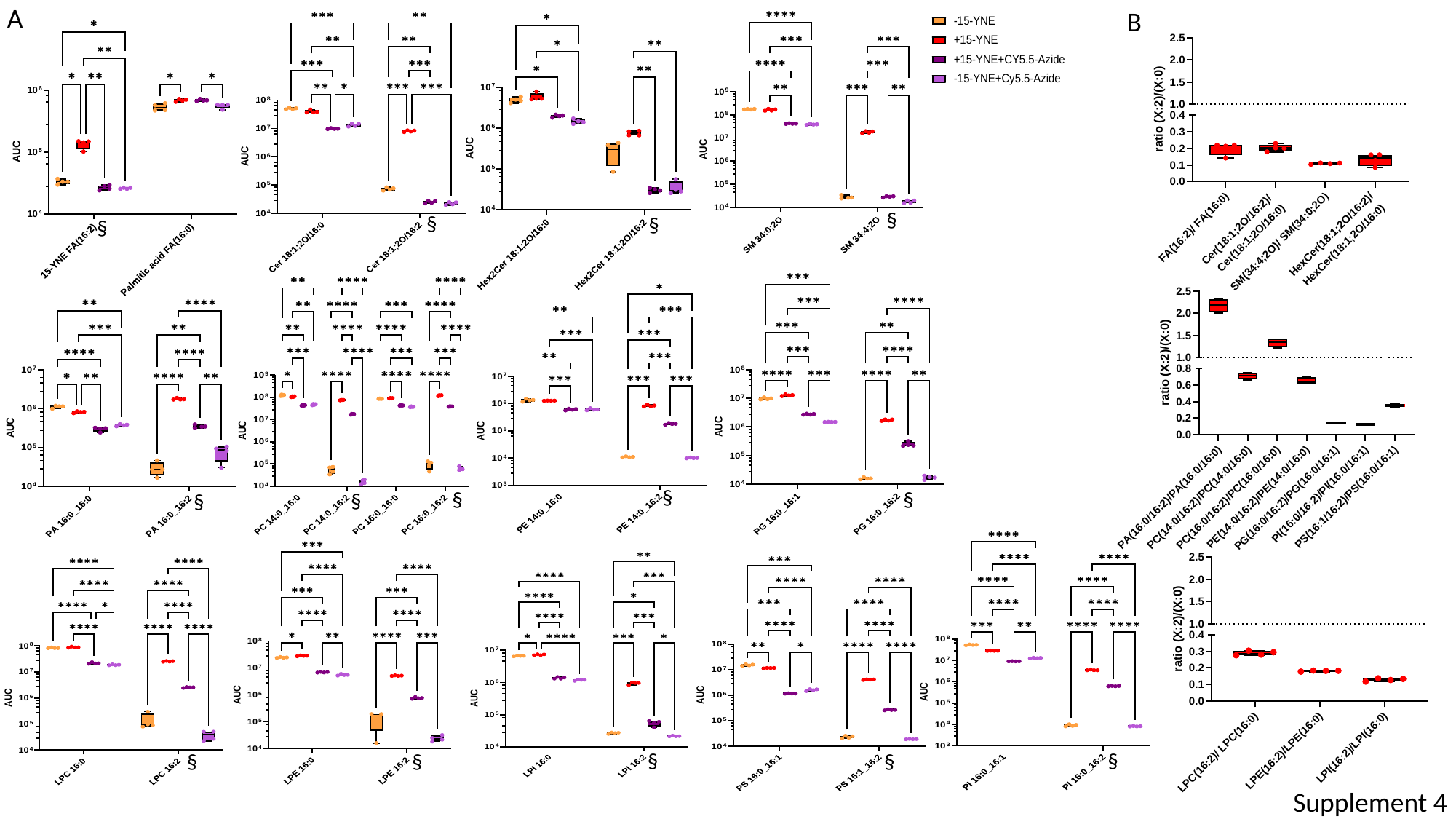

A
B
§
§
§
§
§
§
§
§
§
§
§
§
§
§
Supplement 4

## Slide 2
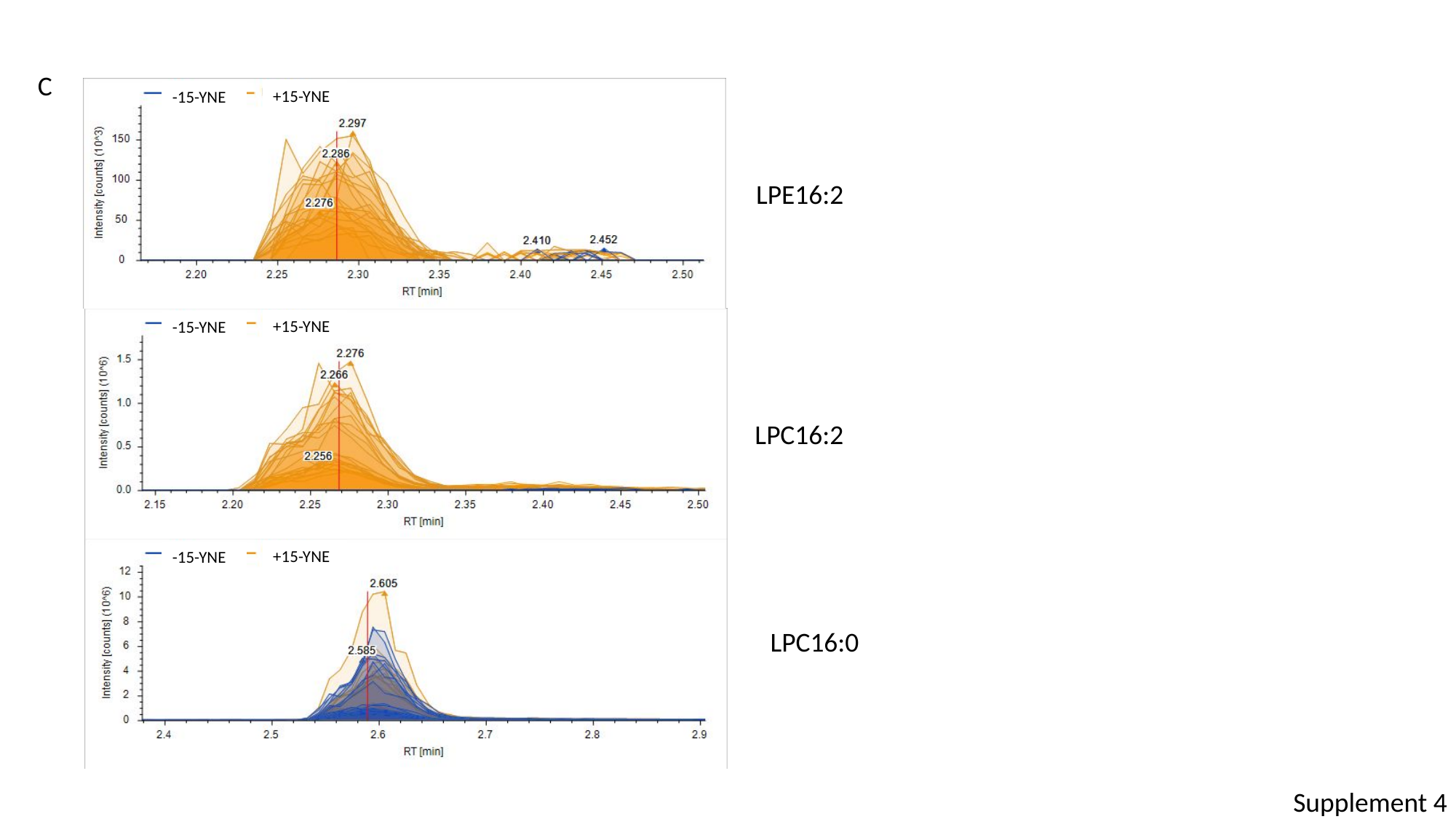

C
+15-YNE
-15-YNE
LPE16:2
+15-YNE
-15-YNE
LPC16:2
+15-YNE
-15-YNE
LPC16:0
Supplement 4

Supplement: Supplementary file 1 [file molecules-30-00377-s001.zip › Supplement 4.pptx]

## Slide 1
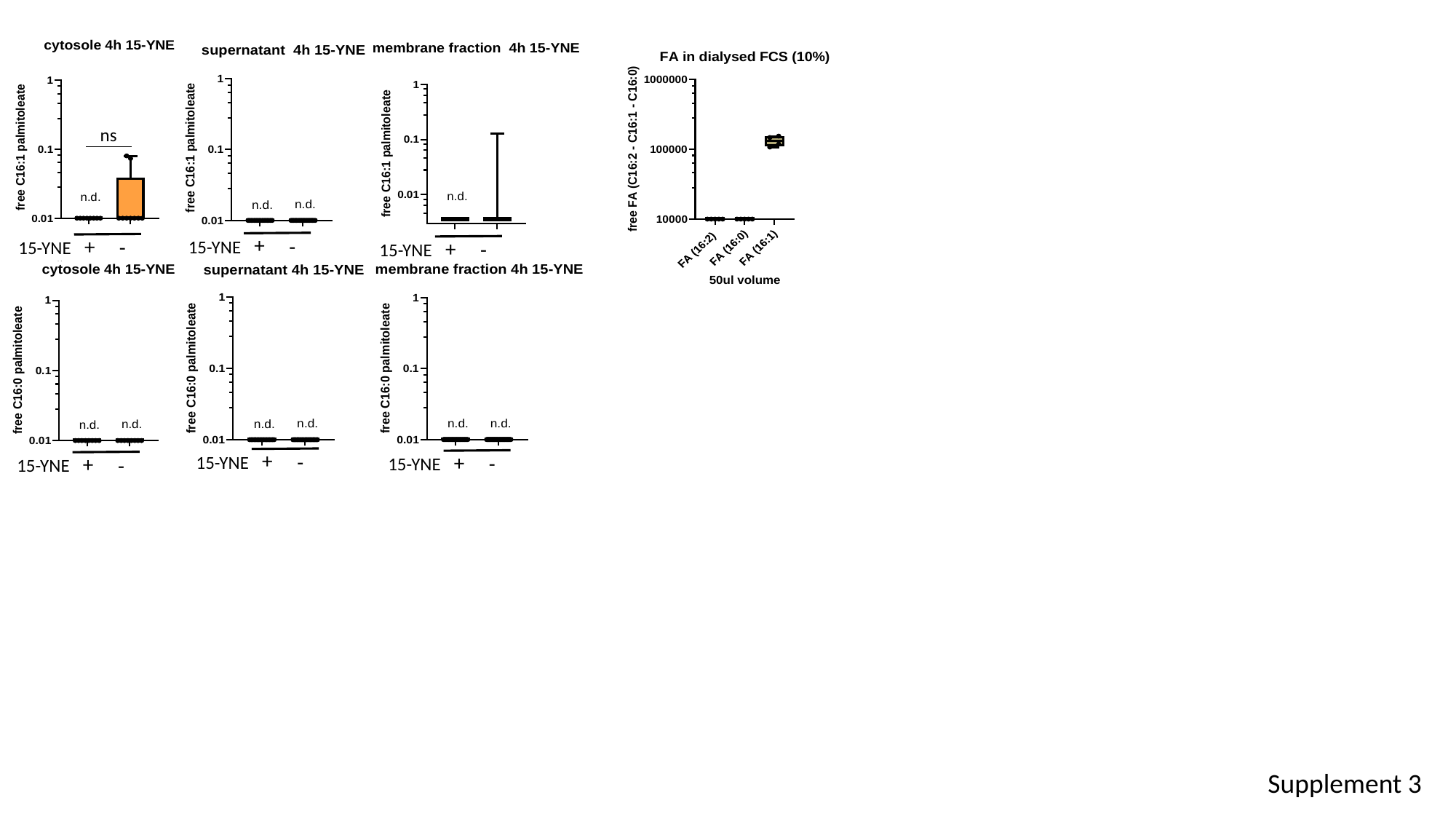

ns
+
-
15-YNE
+
-
15-YNE
+
-
15-YNE
+
-
15-YNE
+
-
15-YNE
+
-
15-YNE
Supplement 3

Supplement: Supplementary file 1 [file molecules-30-00377-s001.zip › Supplement 3.pptx]
